# Are Peer Reviewers Encouraged to Use Reporting Guidelines? A Survey of 116 Health Research Journals

**DOI:** 10.1371/journal.pone.0035621

**Published:** 2012-04-27

**Authors:** Allison Hirst, Douglas G. Altman

**Affiliations:** The EQUATOR Network, Centre for Statistics in Medicine, University of Oxford, Oxford, United Kingdom; University of Ottawa, Canada

## Abstract

**Background:**

Pre-publication peer review of manuscripts should enhance the value of research publications to readers who may wish to utilize findings in clinical care or health policy-making. Much published research across all medical specialties is not useful, may be misleading, wasteful and even harmful. Reporting guidelines are tools that in addition to helping authors prepare better manuscripts may help peer reviewers in assessing them. We examined journals' instructions to peer reviewers to see if and how reviewers are encouraged to use them.

**Methods:**

We surveyed websites of 116 journals from the McMaster list. Main outcomes were 1) identification of online instructions to peer reviewers and 2) presence or absence of key domains within instructions: on journal logistics, reviewer etiquette and addressing manuscript content (11 domains).

**Findings:**

Only 41/116 journals (35%) provided online instructions. All 41 guided reviewers about the logistics of their review processes, 38 (93%) outlined standards of behaviour expected and 39 (95%) contained instruction about evaluating the manuscript content. There was great variation in explicit instruction for reviewers about how to evaluate manuscript content. Almost half of the online instructions 19/41 (46%) mentioned reporting guidelines usually as general statements suggesting they may be useful or asking whether authors had followed them rather than clear instructions about how to use them. All 19 named CONSORT for reporting randomized trials but there was little mention of CONSORT extensions. PRISMA, QUOROM (forerunner of PRISMA), STARD, STROBE and MOOSE were mentioned by several journals. No other reporting guideline was mentioned by more than two journals.

**Conclusions:**

Although almost half of instructions mentioned reporting guidelines, their value in improving research publications is not being fully realised. Journals have a responsibility to support peer reviewers. We make several recommendations including wider reference to the EQUATOR Network online library (www.equator-network.org/).

## Introduction

The medical literature is plagued by poor reporting of research studies hindering its utilisation in clinical practice and further research. This is unethical, wasteful of scarce resources and even potentially harmful [1], [2].

Since the early 1990s, groups consisting primarily of research methodologists and medical journal editors have developed reporting guidelines as tools to help improve the quality of reporting in health research papers. Usually in the form of a checklist, flow diagram and/or explicit text, reporting guidelines specify the essential items required for a clear and transparent account of what was done and what was found in a research study, focusing on issues that might introduce bias into the research. The most widely recognized guidelines are where possible, based on empirical evidence and reflect consensus opinion of experts in a particular field. Reporting guidelines complement generic advice on scientific writing and journals' own specific instructions to authors. Such guidelines include CONSORT (CONsolidated Standards Of Reporting Trials) [3] and PRISMA (Preferred Reporting Items for Systematic reviews and Meta-Analyses) [4]. Almost 200 different reporting guidelines are now catalogued on the EQUATOR (Enhancing the QUAlity and Transparency Of health Research) Network's Library for Health Research Reporting (http://www.equator-network.org/resource-centre/library-of-health-research-reporting/) (accessed 25th October 2011).

Although initial evaluations of reporting guidelines have found that their use is associated with modest improvements in the quality of reporting [5], [6], [7] there has been a lack of awareness of their existence and utility. There are signs that this is improving as authors are increasingly being instructed to follow and complete reporting guideline checklists when submitting manuscripts to journals [8]. A good example of this practice is shown in Box S1.

Peer review is primarily seen as a way to improve the quality of published research reports by filtering out “bad work” and is widely viewed as a “seal of approval” that certain standards have been met, particularly for non-expert readers [9]. “Sense About Science”, a UK charity seeking to promote public understanding of scientific evidence, describes peer review as the “essential arbiter of scientific quality” (www.senseaboutscience.org.uk/index.php/site/project/29/) (accessed 20th October 2011). However, despite this aspiration the ubiquitous use of pre-publication peer review has failed to eliminate errors, inconsistencies and methodological weaknesses in all areas of published medical research [10]. Peer review has been described as “… a flawed process, full of easily identified defects with little evidence that it works” [11]. Despite its shortcomings peer review in principle remains the best method of accrediting publications of health research. A recent UK government inquiry into the current peer review system concluded: “Peer review in scholarly publishing, in one form or another, is crucial to the reputation and reliability of scientific research.…The process, as used by most traditional journals prior to publication, is not perfect, and it is clear that considerable differences in quality exist. However, despite the many criticisms and the little solid evidence on its efficacy, editorial peer review is considered by many as important and not something that can be dispensed with.” It also suggests “There is much that can be done to improve the quality of pre-publication peer review across the board and to better equip the key players to carry out their roles”. (www.publications.parliament.uk/pa/cm201012/cmselect/cmsctech/856/856.pdf) (accessed 28th July 2011).

Peer review informed by reporting guidelines could improve the completeness of information provided in reports of research. Knowing that manuscripts will be assessed using reporting guidelines may also enhance their use by authors when writing their research report thus raising the quality of manuscripts submitted to journals. This in turn may ease their review and hasten the review process.

The primary aim of our study was to assess current practice regarding the provision and content of journals' instructions for peer reviewers of submitted manuscripts, particularly the extent to which reviewers are encouraged or required to use reporting guidelines. A secondary aim was to review the journals' publishers' websites to examine whether any online resources were provided for peer reviewers.

## Methods

### Literature search

In July 2010, we carried out a basic PubMed search for any literature reporting a survey of journals' instructions to peer reviewers and their inclusion of reporting guidelines. We identified none. Following completion of data extraction we carried out a more comprehensive literature search (in April 2011, updated in November 2011), to identify any similar studies with which to compare our results. We searched Embase, PubMed and Cochrane Methodology Register databases. Search terms included MeSH headings for editorial policies, guidelines as topic, peer review, publication/standards, publishing/standards, periodicals as topic/standards, editorial, authorship, and free-text terms for requirement, instruction, policy, guideline, standard, recommendation, author, reviewer, contributor, journal, peer reviewer, editor, and individual reporting guideline acronyms. We still identified no previous study directly examining this issue.

### Reporting of study

We attempted to report this study according to an appropriate reporting guideline but are not aware of one of direct relevance for this type of study. STROBE is designed for epidemiological studies. We consulted a recent overview of guidance on reporting survey research [12] and ensured we reported applicable items in this report.

### Journal sample

We considered several approaches to identifying a useful sample of journals for the survey. Previous methodological research has used random selections of journals, top/highest impact factor journals in general medical or various medical specialty journals, or pre-existing samples such as PubMed “core” journals or the McMaster list. Each sampling method has its flaws when considering generalizability of results. We elected to use the “McMaster list” of journals representing a pre-existing, stable list of publications that are widely used and recommended by clinical practitioners in human healthcare and reviewed by ACP (American College of Physicians) Journal Club (http://hiru.mcmaster.ca/hiru/journalslist.asp). When accessed on 1st September 2010 this list contained 120 titles covering disciplines of medicine, nursing, and occupational and physical therapy. We were interested in journals receiving manuscripts reporting original research. Four of the McMaster list publications did not fit this category and were excluded. Our final journal sample comprised 116 journals (Table S1).

### Survey of availability and content of health research journals' instructions to peer reviewers

Due to limited resources only one author (AH) extracted data. However, a standardised approach to data collection for each journal was used. AH examined the freely accessible areas of all 116 journals' websites between 29th September 2010 and 8th April 2011. Information relating to each journal was extracted from various web pages and collated in a project database (Microsoft Office Excel 2007). We recorded details about the journals' publisher, affiliation with any professional society, whether a general or specialty journal, number of issues per year, whether a member of the Committee on Publication Ethics (COPE) (www.publicationethics.org/), journal impact factor (2009 Thomson Reuters), editor contact details and name of online manuscript submission system. Journals' online instructions to peer reviewers were retrieved and saved both in a print and electronic format if provided. We noted whether, in addition to the core “instructions” text or document, any other form of online guidance was provided. This might include reference to a journal editorial, articles, or slide presentations.

Some journals may provide their instructions to peer reviewers directly rather than openly online. To obtain and examine the content of such instructions we sent an email to the editor-in-chief and/or managing editor of all journals in June 2011. The email described the study and requested details of any “direct to reviewer” guidance. We sent only one request for this information to avoid unduly harassing editors and previous experience by one author (DGA) suggests further requests yield few additional responses.

### Analysis of instructions to peer reviewers

**Figure 1:**
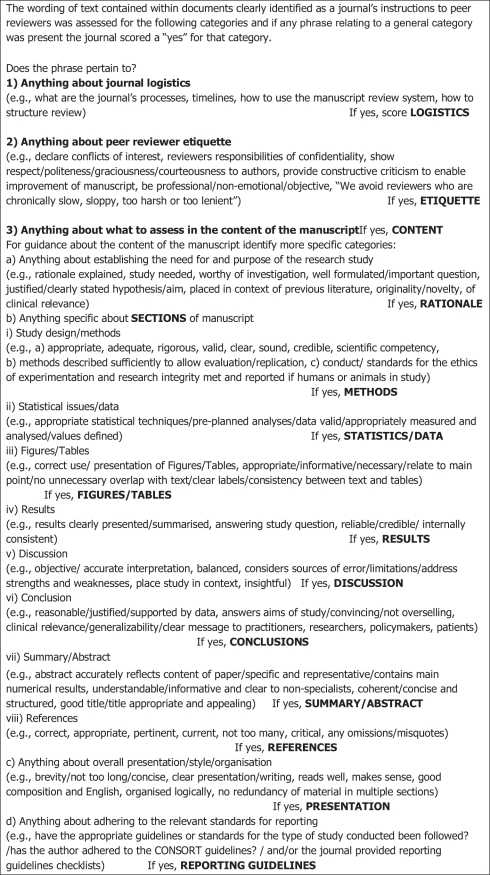
Classification of the text^a^ contained within journal instructions to peer reviewers.

For all online “instructions to (peer) reviewers” retrieved either as text in a webpage or as a separate text document, their content was coded by one author (AH) according to the presence or absence of key domains (Figure 1). The coding of these domains was built on a similar format used in a previous study by one author (DGA) [13] and evolved further through discussion by both authors and review of examples of instructions to peer reviewers known to DGA. We categorised content into three main domains: guidance about journal logistics, about peer reviewer etiquette, and about what to assess in the content of the manuscript. Manuscript content was further divided into 11 sub-sections (Figure 1). We defined a mention of reporting guidelines broadly to include use of a generic term such as “reporting guidelines” or “reporting standards” or specific mention of an individual reporting guideline listed on the EQUATOR website (www.equator-network.org/resource-centre/library-of-health-research-reporting/) or any reference to the EQUATOR Network as a source of information about reporting. When reporting guidelines were mentioned in the instructions to peer reviewers the exact wording used was recorded.

Instructions provided directly to peer reviewers that we received from editors were coded in exactly the same way as the online instructions (Figure 1).

### Publishers' online resources for peer reviewers

We reviewed the websites of all publishers of sample journals to identify any generic resources freely provided for peer reviewers. We examined whether individual journals clearly pointed reviewers to these publisher resources.

### Comments from editors about reporting guidelines

In our email contact with all editors we invited them to share any comments on their experiences or thoughts about using reporting guidelines during peer review. This was an open-ended exploratory invitation and any text received from editors by email was collated thematically.

## Results

### Characteristics of journals

All 116 journals in the sample had a website. Eighty-one of the journals (70%) were produced by commercial publishers while professional societies served as publisher for the remaining 35 (30%). Seventy-four of the 116 journals (64%) were affiliated with one or more professional societies and 82 (71%) were members of COPE (as listed on the COPE website).

Sixteen of the 116 journals (14%) were general medical journals and the rest specialty medical journals. The most frequently represented clinical specialties were orthopaedics (13 journals), nursing (10), clinical neurology (9), cardiac and cardiovascular systems (7), surgery (6) and anaesthesiology, endocrinology and metabolism, paediatrics, rheumatology (each with 5 journals).

The number of journal issues published per year ranged from 4–52 with a median of 12 issues per year (IQR 12–12). The sample journal impact factors ranged from 0.87 to 47.05 (2009 Journal Citation Report (Thomson Reuters, 2010)). Median impact factor was 3.65 (IQR 2.50–6.23). All but two of the journals used an online manuscript submission system for processing manuscripts.

### Availability and content of health research journals' instructions to peer reviewers

#### Online instructions

All 116 journals had websites but only 41 (35%) provided openly accessible online instructions to peer reviewers. All 41 of these instructed peer reviewers about logistics e.g., what are the journal's processes and timescales, how to use the electronic manuscript review system, how to structure the review. Nearly all, 93% (38/41), described standards of behaviour expected from peer reviewers e.g., to declare any conflicts of interest, to uphold the responsibility for confidentiality, to show respect/politeness/graciousness/courtesy to authors, to aim for constructive criticism to enable the improvement of manuscripts, to be professional/non-emotional/objective. One journal stated “We avoid reviewers who are chronically slow, sloppy, too harsh or too lenient”.

Ninety-five per cent (39/41) of the journals' online instructions contained explicit directions to reviewers about assessing one or more aspects of the content of the manuscript (Table 1). Most of the important sections of an original research report were addressed to some degree in these online instructions to peer reviewers. More than 80% of the 41 journals asked reviewers to consider the rationale for the study, methods, statistics/data, results, discussion and conclusion, and also emphasised issues about general presentation, however the level of detail and direction provided varied greatly (Box S2). Some manuscript sections were less likely to be addressed in the online instructions to peer reviewers, but were still mentioned by more than half of the 41 journals. These were how to review figures/tables, summary/abstracts, and references (Table 1). Again the level of detail varied widely between journals (Box S2).

**Table 1:** How often domains were addressed in online instructions to peer reviewers.

Domain	Number of journals (/41) a
**Journal logistics**	41 (100%)
**Peer reviewer etiquette**	38 (93%)
**Manuscript content**	39 (95%)
Rationale	38 (93%)
Methods	38 (93%)
Statistics/Data	35 (85%)
Figures/Tables	28 (68%)
Results	35 (85%)
Discussion	34 (83%)
Conclusion	37 (90%)
References	24 (58%)
Summary/Abstract	26 (63%)
General Presentation	37 (90%)
Reporting guidelines	19 (46%)

a Obtained from surveying 116 journal websites Sept 2010-April 2011. Only 41 of the 116 journals' websites provided online instructions for peer reviewers.

Reporting guidelines were mentioned in almost half of the online instructions to peer reviewers 19/41 (46%). These tended to be in the form of general references or statements about reporting guidelines or standards suggesting they may be useful to the peer reviewer or asking in general whether the author has followed them rather than explicit instructions about exactly how to use them in the peer review process. One journal, *Nursing Research*, did instruct peer reviewers to use the CONSORT checklist (Box S3).

All of the 19 journals that mentioned reporting guidelines named CONSORT, the guidance for reporting randomized controlled trials (RCT). Twelve of these provided the URL for the CONSORT website (www.consort-statement.org/), five referenced the superseded 2001 publication only [14] and two provided no reference at all. Several extensions to the CONSORT Statement have been developed in response to poor reporting of specific issues relating to particular trial designs, interventions or data types (www.consort-statement.org/extensions/). Reference to these extensions was rare, only two journals mentioned CONSORT for abstracts [15] and two the extension for non-pharmacological treatments [16].

With regard to reporting guidance for systematic reviews of RCTs, QUOROM [17] and PRISMA [4] (www.prisma-statement.org/) were each mentioned by four journals. PRISMA superseded QUOROM in 2009 so consequently four journals were out of date in their guidance identified in the six months up to April 2011. If we consider both guidelines together then an acknowledgement of a reporting guideline for reporting systematic reviews of RCTs was identified for peer reviewers by eight journals (42%).

STARD [18] (www.stard-statement.org/) for reporting of studies of diagnostic accuracy was mentioned in five journals. Reporting guidance for observational studies STROBE [19] (www.strobe-statement.org/) was referenced by four journals and MOOSE for meta-analyses of observational studies [20] by six journals. Other reporting guidelines received only one or two mentions: TREND [21], SQUIRE [22], RATS [23], COREQ [24], QUALRES [25], biomedical images [26]. Similarly, the EQUATOR Network, which provides a free, up to date online library of all reporting guidelines, was highlighted by only two journals in their peer reviewer instructions as a useful resource for reviewers of manuscripts (Table 2).

**Table 2:** How often individual reporting guidelines (RG) appeared in online instructions to peer reviewers (n = 19).

Reporting Guideline (RG)	No. of journals mentioning RG (/19)	URL
**CONSORT** for RCTs	19 (100%)	www.consort-statement.org/
**CONSORT extensions**		www.consort-statement.org/extensions/
Abstracts	2 (10%)	
Non-pharmacological interventions	2 (10%)	
Others (e.g., cluster, non-inferiority, pragmatic trials, herbal, acupuncture, harms)	None	
**QUOROM** for systematic reviews of RCTs	4 (21%)	
**PRISMA** for systematic reviews of RCTs (replaces QUOROM since 2009)	4 (21%)	www.prisma-statement.org/
**STARD** for diagnostic accuracy studies	5 (26%)	www.stard-statement.org/
**STROBE** for observational studies	4 (21%)	www.strobe-statement.org/
**MOOSE** for meta-analysis of observational studies	6 (32%)	
**TREND** for nonrandomized designs	2 (10%)	www.cdc.gov/trendstatement/
**SQUIRE** for quality improvement	2 (10%)	www.squire-statement.org/
**RATS** for qualitative research	1 (5%)	www.biomedcentral.com/info/ifora/rats
**COREQ** for qualitative research	1 (5%)	
**QUALRES** for qualitative research	1 (5%)	www.qualres.org/
**Images** for biomedical images	1 (5%)	Reference 26
**EQUATOR** Network	2 (10%)	www.equator-network.org/

**Abbreviations:**

CONSORT – CONsolidated Standards Of Reporting Trials.

QUOROM – The QUality Of Reporting Of Meta-analyses of randomised trials.

PRISMA – Preferred Reporting Items for Systematic reviews and Meta-Analyses.

STARD – STAndards for Reporting of Diagnostic accuracy.

STROBE – STrengthening the Reporting of Observational studies in Epidemiology.

MOOSE – Meta-analysis Of Observational Studies in Epidemiology.

TREND – Transparent Reporting of Evaluations with Non-randomised Designs.

SQUIRE – Standards for Quality Improvement Reporting Excellence.

RATS – Qualitative research review (Relevance, Appropriateness, Transparency, Soundness).

COREQ – COnsolidated criteria for REporting Qualitative research.

QUALRES – QUAlitative RESearch.

EQUATOR – Enhancing the QUAlity and Transparency Of health Research.

RCT – randomized controlled trial.

#### Instructions provided directly to individual reviewers

Twenty-seven (23%) of the 116 journal editors responded to our email. Nineteen editors confirmed the availability of their online instructions already obtained and did not provide us with any additional information. Eight editors of journals that did not provide online instructions supplied us with the instructions they email directly to reviewers following acceptance of the invitation to review. These instructions were coded as detailed in Figure 1. All eight addressed journal logistics, only three outlined peer reviewer etiquette but all eight considered the content of the manuscript. Specific sections mentioned were as follows: “Rationale” and “methods” (by eight journals), “statistics/data” (by seven), “figures/tables” and “references” (by six), “discussion” and “summary/abstract” (by four), “conclusion” (by two), six asked about “general presentation” and two mentioned “reporting guidelines”.

### Additional online information provided by journals for peer reviewers

Further information was provided online by 19 (46%) journals. Eleven cited articles about peer review published in their own or other journals. These included Archives of Pediatric and Adolescent Medicine [27] and the Journal of the American College of Cardiology [28]. Six provided slide presentations with or without audio/video. For example the Journal of Vascular Surgery provides an online video presentation, “How to Review A Scientific Paper for JVS: A View from the Editors' Desk (presented at the 2008 Vascular Annual Meeting) (www.jvascsurg.org/).

### Publishers' online resources for peer reviewers

We found useful web resources for reviewers from four publishers of journals in this survey: Nature Publishing Group (www.nature.com/authors/peer_review/index.html), Elsevier (www.elsevier.com/wps/find/reviewershome.reviewers), BMJ Publishing Group Ltd (www.resources.bmj.com/bmj/reviewers) and Wiley-Blackwell (for its nursing journals) (www.nurseauthoreditor.com/forreviewers.asp) (all accessed during March 2011). All provided information about the processes of peer review, the purpose and history of peer review and encouraged debate and research about various methods of peer review. Individual journals did not routinely direct reviewers to their publishers' resources from their instructions to peer reviewers. However the specific practice depended largely on use of manuscript submission systems. For example, Elsevier published 25 of the journals in our sample. Eighteen of these journals directed peer reviewers to Elsevier reviewers' resources from within the Elsevier Editorial System (EES). The seven Elsevier journals that did not use EES did not alert reviewers to Elsevier's resources.

### Editors' comments about use of reporting guidelines in peer review

Only five of the 116 editors took up our invitation to comment about their experience of using reporting guidelines in the peer review process so we were not able to comprehensively investigate this question. The comments we did receive reflected a diverse range of attitudes and practices. Following our contact one journal immediately changed practice and incorporated reporting guidelines in their online reviewer instructions. One reported they already had a solid system in place to routinely make use of reporting guidelines. One did have policies about their use but felt under-resourced to enforce them and two implied there was no real need for them to consider their use in this way.

## Discussion

### Provision of instructions to peer reviewers

All 116 journals included in this survey had their own website so could have made their instructions to peer reviewers openly accessible online. This would improve transparency of their review processes and tell authors of manuscripts what peer reviewers will assess in their paper. However, only 41 (35%) of the journals provided their instructions to peer reviewers in this way. We suggest that all journals take this simple step towards transparency.

### Content of journals' instructions to peer reviewers

The majority of journals' instructions to peer reviewers included guidance about journal logistics, peer reviewer etiquette and the content of the manuscript (usually sub-divided by the main IMRAD style sections of a research paper). However, the level of detail and explicit instruction varied greatly across journals. There is not one universal standard “consensus” set of instructions for peer reviewers akin to the “Uniform Requirements for Manuscripts Submitted to Biomedical Journals” developed by the International Committee of Medical Journal Editors (http://www.icmje.org/urm_full.pdf) (accessed 21st Oct 2011) for authors of research papers. Whether such a notion is desirable or feasible, and which body might oversee its development, is an open question. Others have proposed this idea previously. Frank [29] reviewed what editors requested of peer reviewers in 73 US-based journals in 1992 (prior to reporting guidelines). She concluded that journals varied substantially in their requests and suggested several areas that could be standardised to improve the process. More recent initiatives to assemble very comprehensive generic instructions for peer reviewers have also included a role for reporting guidelines [30], [31]. The article published by Elsevier España, S.L. on behalf of Sociedade Portuguesa de Pneumologia [31], provides a helpful “checklist for the assessment of manuscript quality” and highlights that “in addition to these general questions it is very helpful to use specific checklists available to assess each study design. The EQUATOR Network keeps updated resources on checklists and guidelines on reporting medical research literature”. Another freely available online resource for health professionals who are serving, or wish to serve, as peer reviewers of the biomedical literature is “Translating Critical Appraisal of a Manuscript into Meaningful Peer Review”, provided by the Cochrane Eyes and Vision Group (http://eyes.cochrane.org/).http://trams.jhsph.edu/trams/index.cfm?event=training.launch&trainingID=132 (accessed 25th October 2011).

### Reporting guidelines in journals' instructions to peer reviewers

Around half (46%) of the 41 sets of instructions to peer reviewers that we accessed from journal websites in this survey mentioned reporting guidelines, suggesting the potential value of these tools is not being fully realised. This underuse may stem from three factors.

First, there may have been a lack of awareness of their existence. Many organisations are now helping to raise awareness of these tools. These include the US National Library of Medicine guide (http://www.nlm.nih.gov/services/research_report_guide.html) (accessed 23rd February 2012) and the UK General Medical Council (GMC) “Good practice in research” document. (http://www.gmc-uk.org/guidance/ethical_guidance/6005.asp) (accessed 27th February 2012). Some funding bodies now include reference to reporting guidelines in their investigator resources, e.g. UK NIHR HTA “Resources for Authors” (http://www.hta.ac.uk/investigators/rfa.pdf) (accessed 23rd February 2012) and the UK Medical Research Council (MRC) “MRC good research practice: principles and guidelines” document (January 2012 draft for consultation) (http://www.mrc.ac.uk) (accessed 27th February 2012) which includes the statement “G.7 Agreed standards, such as the CONSORT Statement (CONsolidated Standards of Reporting Trials), and the ARRIVE guidance (Animal Research: Reporting in-vivo experiments) should be observed.”

Second, there may be uncertainty as to their utility to improve research articles (both generically and for individual guidelines). Research in this field is ongoing but currently there is some evidence that introducing CONSORT within journals is associated with improved quality of reports of RCTs [3], [5], [7] and similarly for STARD [6]. There is also some evidence that introduction of a 23-item reporting checklist for authors by a journal improved reporting quality in non-randomised paediatric surgical studies [32].

Third, there is the issue of exactly how to use reporting guidelines in the peer review process. A few randomised trials have been conducted comparing strategies for peer review which have included reporting guidelines. Cobo and colleagues [33] compared the effects on manuscript quality of either adding a statistical peer reviewer or suggesting the use of reporting checklists to clinical reviewers or both. They concluded “This prospective randomized study shows the positive effect of adding a statistical reviewer to the field-expert peers in improving manuscript quality. We did not find a statistically significant positive effect by suggesting reviewers use reporting guidelines”. A more recent trial by the same authors [34] assessed additional peer review using reporting guidelines compared with conventional peer review alone in 92 manuscripts reviewed from May 2008 to April 2009 for the journal *Medicina Clinica* (which did not mention reporting guidelines in its instructions to authors). Authors received feedback from reviewers and the quality of their manuscript was assessed before and after responding to reviewers' comments using the Goodman Scale [35]. Their findings were suggestive, but not conclusive, that peer review using reporting guidelines can improve the study report quality more than not using them. Further research needs to be undertaken to establish the most effective methods of using reporting guidelines in peer review. It is important to recognise that there is potential for abuse of reporting guidelines [36] and they should not be used by peer reviewers and editors as critical appraisal checklists to reject manuscripts. Groves [37] clarifies the role of reporting guidelines as follows: “Editors should not, however, use these reporting guidelines to reject studies that do not reach some fixed or arbitrary threshold for quality. In difficult and new areas of research, imperfectly conducted studies often provide good enough evidence to change policy or practice or to inform the next phase of research. Such studies deserve to be published, warts and all, but reporting guidelines point out where the warts are and how big they are.”

### Additional online information provided by journals for peer reviewers

Nearly half (19/41) of the journals in our survey that provided online instructions also used additional formats for informing peer reviewers. However, it is not known which methods or formats may be best for educating reviewers [38]. Further research may help to identify successful methods.

### Publishers' online resources for peer reviewers

The larger publishers of journals in this survey had prepared online resources for peer reviewers but there was inconsistent linkage between journals and these resources. More journals could direct peer reviewers to generic resources provided on publishers' websites. A survey of peer reviewers might inform how useful they find the information provided online by journals and publishers.

### Implications for practice: How journal editors and publishers might help peer reviewers

Peer reviewers are volunteers and difficult to recruit due to high workloads and competing time pressures [39], factors predicting good peer reviewers are elusive [40], and the performance of peer reviewers tends to deteriorate gradually [41]. Recognition of continuing peer reviewer development in academic environments could help in all these areas. One international study of peer reviewers for nursing journals [42] identified an unmet need expressed by reviewers for more training and feedback.

Journal editors and publishers have an ethical obligation to support peer reviewers to strive for transparent and accurate reporting of research. Over 70% of the journals in this sample were members of COPE which clearly identifies this responsibility in its Codes of Conduct for journal editors and publishers (www.publicationethics.org/resources/code-conduct) (accessed 12^th^ December 2011). The EQUATOR Network has previously formulated a number of recommendations for actions that journals and publishers might take to help improve the quality of published health research [1]. We add further recommendations regarding peer review (Box S4). These include encouraging editors to write editorials about reporting guidelines [43], [44], [45], [46], [47], [48], [49].

### Limitations of study

Our survey provides a snapshot of the availability and content of health journals' instructions to peer reviewers particularly with regard to reporting guidelines. We recognise that the survey has several limitations. We acknowledge that identifying a method to select a sample of journals for this type of study is problematic. Our sample was relatively small (n = 116) sample drawn from the McMaster list. These were mostly “traditional” journals “selected based on suggestions by librarians, clinicians, editors, and editorial staff, Science Citation Index (SCI) impact factors; systematic examination of the contents of each selected journal for at least 6 months; and by ongoing yield of articles that meet basic inclusion criteria for assessing the quality of studies concerning the cause, course, prediction, diagnosis, prognosis, prevention, and treatment of medical disorders” (http://hiru.mcmaster.ca/hiru/HIRU_McMaster_PLUS_Projects.aspx) (accessed 27th February 2012). Some of the newer and more innovative open access publishers, including PLoS (www.plos.org/), BMC (www.biomedcentral.com/) and BMJOpen (www.bmjopen.bmj.com/) are not currently represented in the McMaster list. These journals may be more likely to advocate the use of reporting guidelines. For example, “*BMC Pediatrics* supports initiatives aimed at improving the reporting of biomedical research. We recommend authors refer to the EQUATOR network website for further information on the available reporting guidelines for health research, and the MIBBI Portal for prescriptive checklists for reporting biological and biomedical research where applicable. Authors are requested to make use of these when drafting their manuscript and peer reviewers will also be asked to refer to these checklists when evaluating these studies”. http://www.biomedcentral.com/bmcpediatr/about#reporting (accessed 12^th^ December 2011). Our study sample is, however, a reasonable sample of journals (both general medical and specialty) that are widely read by clinical practitioners and would therefore be expected to be aiming to maximise the quality and utility of their articles.

Other limitations relate to having only a single data extractor but we standardised the data extraction process to maximise consistency. Similarly we were only able to have one author code the content of reviewer instructions. However this followed a lengthy coding development process involving the consensus of both authors.

The website information was extracted in the six months prior to April 2011 and some journals may have since updated their websites and their instructions to peer reviewers.

### Conclusions

Traditional pre-publication peer review of manuscripts submitted to journals is a complex process. A large burden of responsibility falls on the shoulders of busy unpaid reviewers who may not be fully equipped to carry out the role. While its many flaws are widely acknowledged, peer review is here to stay and we must turn our attention to constructive ways of improving the process. It is likely this will require a multi-dimensional approach, including training of peer reviewers.

Reporting guidelines used appropriately are an important tool to improve the value of published research to users and their potential is not currently being fully realised. We suggest actions that journals and publishers could take to increase awareness of and fully utilise reporting guidelines in their peer review process (Box S4). If more journals and publishers followed these recommendations the value of publications and of health research itself might improve and be less wasteful.

## Supporting Information

Box S1 **Example of instructions to authors: the European Journal of Clinical Investigation.**
(DOCX)

Box S2 **Examples of guidance about peer reviewing manuscript content.**
(DOCX)

Box S3 **Examples of how reporting guidelines were mentioned in journal's instructions to peer reviewers.**
(DOCX)

Box S4 **Recommendations for journal editors: how to improve the peer review of submitted manuscripts.**
(DOCX)

Table S1 **Journals included in survey of instructions to peer reviewers.**
(DOCX)
