# Supplementary material for: Are Peer Reviewers Encouraged to Use Reporting Guidelines? A Survey of 116 Health Research Journals
Source: PLoS One. 2012 Apr 27;7(4):e35621. doi: 10.1371/journal.pone.0035621 (PMC3338712; doi:10.1371/journal.pone.0035621)
Supplement: Box S1 — Example of instructions to authors: the European Journal of Clinical Investigation. (DOCX) [file pone.0035621.s001.docx]

**Box S1. Example of instructions to authors: the European Journal of Clinical Investigation.**

*“Reporting standards for research: The EJCI complies with main reporting standards for key types of research.. as outlined in the EQUATOR website (*[*http://www.equator-network.org*](http://www.equator-network.org)*) and in the EQUATOR article published in the first issue of January 2010 of EJCI .. When a study is of a type that needs to adhere to such EQUATOR-listed standards, the authors should make appropriate citation to them to mention that the reporting is compliant with these standards”.* [*http://www.wiley.com/bw/submit.asp?ref=0014-2972*](http://www.wiley.com/bw/submit.asp?ref=0014-2972) *(accessed 25^th^ October 2011)*
