# Supplementary material for: Are Peer Reviewers Encouraged to Use Reporting Guidelines? A Survey of 116 Health Research Journals
Source: PLoS One. 2012 Apr 27;7(4):e35621. doi: 10.1371/journal.pone.0035621 (PMC3338712; doi:10.1371/journal.pone.0035621)
Supplement: Box S2 — Examples of guidance about peer reviewing manuscript content. (DOCX) [file pone.0035621.s002.docx]

**Box S2. Examples of guidance about peer reviewing manuscript content.**

***Example 1: How to assess rationale for study.***

*• “Does it state the problem and study objectives clearly and adequately?” [J Pediatr]*

*• “A. Internal Validity: Are the research questions identified clearly and logically? Are the research questions/problems/hypotheses clearly stated? Does the introductory discussion of the recent literature logically lead to the research questions under study? Are the research questions placed in a coherent theoretical context? B. External Validity: Is the research question worth asking (i.e., is the problem of concern to nursing)? What is the clinical significance of the research findings? How generalizable are the findings to other situations or populations? Are there problems with confidentiality or other ethical considerations? Does the study contribute to the development of knowledge in nursing?” [Can J Nurs Res]*

*• “Does it explain why the study was done? Is it too long….or too short? Is the research question/hypothesis/objective stated explicitly and correctly? Can you tell why the authors chose this question? Is it the right question? Does the paper pass the “so what” test, especially within the context of our journal and its mission? Begin thinking about the following: What is the importance of this topic to academic EM and to the journal? What is the importance of this study compared to others in the same area? What is the relevance of this topic to the journal’s audience?” [Acad Emerg Med]*

***Example 2: How to assess methods section.***

*• “Are the methods and experimental techniques adequate?” [J Thorac Cardiovasc Surg]*

*• “Are the methods described clearly? (The methods section should in principle allow full replication of the study. Could you follow it? Was it complete? Did you understand what was done? Is there unnecessary discussion?) and, Are the methods used appropriate? (This refers to the design, measures and analytic procedures actually used. Was the design appropriate? Were the measures used or data collected appropriate?)” [Clin Rehabil]*

***Example 3: How to assess the abstract.***

*• Is it “informative and written in a style that will make it intelligible to readers who do not work in the specific area?” [J Thorac Cardiovasc Surg]*

*• “The abstract should indicate the purpose of the study, subjects and methods used, most important results and the main conclusions, which the data support.” [Ann Thorac Surg]*

***Example 4: How to assess figures/tables.***

*• “What figures/tables could be in color?” [Am J Gastroenterol]*

*•The reviewer should comment on “quality of and need for figures and tables” [J Trauma]*

*• “Tables and Figures: Are there enough illustrations? Too many? Is there overlap with text? Are labels clear? Are important features visible and well-marked? Are legends understandable? Is the quality of the figures adequate for reproduction?” [J Pediatr]*
