# Supplementary material for: Are Peer Reviewers Encouraged to Use Reporting Guidelines? A Survey of 116 Health Research Journals
Source: PLoS One. 2012 Apr 27;7(4):e35621. doi: 10.1371/journal.pone.0035621 (PMC3338712; doi:10.1371/journal.pone.0035621)
Supplement: Box S3 — Examples of how reporting guidelines were mentioned in journal's instructions to peer reviewers. (DOCX) [file pone.0035621.s003.docx]

**Box S3. Examples of how reporting guidelines were mentioned in journal’s instructions to peer reviewers.**

***a) General statements about reporting guidelines being useful to reviewer:***

*• “Reviewer Tools and Resources: The following links may be useful as you review manuscripts submitted to [JOSPT]:” links to CONSORT, QUOROM, STARD, STROBE, MOOSE.*

*• “Reviewers may find the checklists (which authors are required to submit with their papers) helpful as a reference when they are reviewing a manuscript.” (lists CONSORT, STROBE, STARD, QUALRES and PRISMA). [Phys Ther]*

***b) Questions about whether the authors have followed reporting guidelines:***

*• “Have the appropriate guidelines or standards for the type of study conducted been followed (e.g., CONSORT criteria for randomized trials)?” [Am J Gastroenterol]*

*• “For randomised trials, systematic reviews, observational studies, health economics studies – (are they) reported in line with the appropriate reporting statement or checklist? (eg CONSORT, PRISMA, MOOSE, STROBE) Do these contain information that should be better reported in the manuscript, or raise questions about the work?” [BMJ]*

*• “Referees are asked to evaluate whether the manuscript: - Has been written following the CONSORT checklist and the CONSORT flow diagram provided” [Pain]*

***c) Instructions to actively check that items in reporting guidelines are reported:***

*• “If the manuscript is based on RCT design, it is necessary to assess the manuscript for the adequacy of steps taken to control bias. We encourage reviewers to visit http://www.consort-statement.org and to use the CONSORT checklist with specific attention to items that if inadequately reported are associated with biased estimates of treatment effect as well as information essential to evaluate the reliability and relevance of the results. The CONSORT Website is updated frequently and to assure that you are using current information it is important that you visit the Web site.” [Nurs Res]*
