# Supplementary material for: Are Peer Reviewers Encouraged to Use Reporting Guidelines? A Survey of 116 Health Research Journals
Source: PLoS One. 2012 Apr 27;7(4):e35621. doi: 10.1371/journal.pone.0035621 (PMC3338712; doi:10.1371/journal.pone.0035621)
Supplement: Box S4 — Recommendations for journal editors: how to improve the peer review of submitted manuscripts. (DOCX) [file pone.0035621.s004.docx]

**Box S4. Recommendations for journal editors: how to improve the peer review of submitted manuscripts.**

a) Increase transparency of your peer review process by providing your instructions to peer reviewers openly on your website. Ideally instructions should be collated in one place, made available as a printable pdf and include the date of their last revision. Consider linking to these from your instructions to authors to give your authors an indication of how their manuscript will be evaluated.

b) Alert your peer reviewers to the importance of good reporting and the availability of reporting guidelines which can act as an *aide memoire* for items indicating complete reporting. Provide or link to relevant guidelines/checklists and ask peer reviewers to use them during their manuscript assessment. This will make the review more helpful for authors in revising their manuscripts.

c) If you provide training for peer reviewers consider a module on reporting guidelines and how they can be used in manuscript assessment. Link to other available resources e.g, [30,31], and the Cochrane Eyes and Vision online resource.

d) Consider including a link to the EQUATOR Network (www.equator-network.org) as the portal for up to date reporting guidelines and other related resources. This will ensure that your instructions are current without additional effort for your journal.

e) Publish editorials to widen awareness of the importance of good reporting, the use of reporting guidelines by authors and peer reviewers and indicate your editorial policies that will be incorporating them. A number of journals in a variety of clinical areas have already done this [43,44,45,46,47,48,49] .

f) Where provided, journals should link to resources for peer reviewers provided by their publishers.
