# Supplementary material for: Are Peer Reviewers Encouraged to Use Reporting Guidelines? A Survey of 116 Health Research Journals
Source: PLoS One. 2012 Apr 27;7(4):e35621. doi: 10.1371/journal.pone.0035621 (PMC3338712; doi:10.1371/journal.pone.0035621)
Supplement: Table S1 — Journals included in survey of instructions to peer reviewers. (DOCX) [file pone.0035621.s005.docx]

**Supplementary Table:**

**Table S1. Journals included in survey of instructions to peer reviewers.**

| *Acad Emerg Med*  *Acta Orthop*  *Age Ageing*  *Am J Cardiol*  *Am J Epidemiol*  *Am J Gastroenterol*  *Am J Kidney Dis*  *Am J Med*  *Am J Obstet Gynecol*  *Am J Psychiatry*  *Am J Public Health*  *Am J Respir Crit Care Med*  *Am J Sports Med*  *Anesth Analg*  *Anesthesiology*  *Ann Emerg Med*  *Ann Fam Med*  *Ann Intern Med*  *Ann Neurol*  *Ann Rheum Dis*  *Ann Surg*  *Ann Thorac Surg*  *Appl Nurs Res*  *Arch Dis Child*  *Arch Dis Child Fetal Neonatal Ed*  *Arch Gen Psychiatry*  *Arch Intern Med*  *Arch Pediatr Adolesc Med*  *Arch Phys Med Rehabil*  *Arch Surg*  *Arthritis Care Res (Hoboken)*  *Arthritis Rheum*  *Arthroscopy*  *BJOG*  *BMJ*  *Br J Gen Pract*  *Br J Psychiatry*  *Br J Surg* | *Can J Anaesth*  *Can J Nurs Res*  *Cancer Nurs*  *Chest*  *Circulation*  *Clin J Am Soc Nephrol*  *Clin J Pain*  *Clin Orthop Relat Res*  *Clin Rehabil*  *CMAJ*  *Crit Care Med*  *Diabet Med*  *Diabetes Care*  *Diabetes Obes Metab*  *Eur Heart J*  *Fam Pract*  *Foot Ankle Int*  *Gastroenterology*  *Gut*  *Headache*  *Heart*  *Int J Clin Pract*  *Int J Nurs Stud*  *Int J Obes (Lond)*  *J Adv Nurs*  *J Am Coll Cardiol*  *J Am Coll Surg*  *J Am Geriatr Soc*  *J Am Soc Nephrol*  *J Arthroplasty*  *J Bone Joint Surg Am*  *J Bone Joint Surg Br*  *J Clin Nurs*  *J Clin Oncol*  *J Fam Pract*  *J Gen Intern Med*  *J Infect Dis*  *J Neurol Neurosurg*  *Psychiatry* | *J Neurosurg*  *J Nurs Scholarsh*  *J Orthop Sports Phys Ther*  *J Orthop Trauma*  *J Pain*  *J Pediatr*  *J Pediatr Orthop*  *J Physiother*  *J Rheumatol*  *J Shoulder Elbow Surg*  *J Surg Oncol*  *J Thorac Cardiovasc Surg*  *J Trauma*  *J Vasc Surg*  *JAMA*  *Kidney Int*  *Lancet*  *Lancet Neurol*  *Lancet Oncol*  *Mayo Clin Proc*  *Med Care*  *N Engl J Med*  *Neurology*  *Nurs Res*  *Obesity (Silver Spring)*  *Obstet Gynecol*  *Pain*  *Pediatrics*  *Pharmacotherapy*  *Phys Ther*  *Prev Med*  *Res Nurs Health*  *Rheumatology (Oxford)*  *Spine*  *Spine J*  *Stroke*  *Thorax*  *Transplantation*  *West J Nurs Res*  *World J Surg* |
| --- | --- | --- |

Four exclusions from the McMaster list accessed 1st September 2010 were:

Canadian Agency for Drugs and Technologies in Health (CADTH), Cochrane Database of Systematic Reviews, Evidence Report -Technology Assessment (AHRQ) and Health Technology Assessment reports (HTA).
